# Association of hip dysplasia in newborns with ethnicity using graf method ultrasound

**DOI:** 10.12669/pjms.41.1.8600

**Published:** 2025-01

**Authors:** Nasim Marvi, Samia Khalid Khokhar, Aisha Qamar, Muhammad Ashfaq

**Affiliations:** 1 Nasim Marvi, MPhil Senior Lecturer, Department of Anatomy, Jinnah Sindh Medical University, Karachi, Pakistan; 2 Samia Khalid Khokhar, MPhil Assistant Professor, Department of Anatomy, Bahria University Health Sciences Campus, Karachi, Pakistan; 3 Aisha Qamar, MPhil Professor, Department of Anatomy, Bahria University Health Sciences Campus, Karachi, Pakistan; 4 Muhammad Ashfaq, MCPS, FCPS, CHPE (JSMU) Pediatric Medicine, National Institute of Child Health Karachi, Pakistan

**Keywords:** Hip Dysplasia, Congenital, Ultrasonography

## Abstract

**Objectives:**

To determine gender and ethnic distribution of developmental dysplasia of hip in newborns using Graf method for ultrasound. To determine the significance of association between developmental dysplasia of hip with ethnicity in newborns using Graf’s method ultrasound.

**Method:**

This analytical cross-sectional observational study was conducted in Karachi from January through June 2022 .A total of 115 healthy newborns (both male and female) aged under 28 days were included in the study after parental informed consent. Hip dislocation was assessed clinically using Barlow and Ortolani maneuvers. The gender, ethnicity and hip angles were recorded in the proforma. Graf’s method ultrasound was used to measure the hip angles. Acetabular roof, head of femur, triradiate cartilage and acetabular labrum were used as landmarks for angle measurements. The hip joint was classified based on Graf method criteria.

**Results:**

The incidence of hip dysplasia was higher in male (16.08%) as compared to female infants (14.78%). The difference between gender distributions was not significant. The predominant ethnicities with pathological hip were Sindhi (9.13%), Punjabi (7.39%) and Pathan (6.08%). The association between ethnicity and pathological hips was statistically significant (p=0.021).

**Conclusion:**

There is no significant difference in incidence of DDH with gender. However, there is a significant association between ethnicity and incidence of developmental dysplasia of hip in neonates of 28 days age. Incidence of DDH is high in the Sindhi, Punjabi and Pathan populations.

## INTRODUCTION

Developmental dysplasia of hip (DDH) represents a range of anatomic hip anomalies that affect hip stability and development.^1^ It is caused by a developmental aberration that results in a defect in the hip ranging from laxity to complete dislocation.^2^

According to the available research, the incidence of DDH in infants significantly changes with geographical location, ranging from its lowest point in populations of Africans to its highest point in populations of Caucasians.^3^ In Pakistan there is a high prevalence of congenital anomalies. A study in Khyber Pakhtunkhwa reported prevalence of 24.6% for limb abnormalities including DDH.^4^ The reported incidence of DDH ranges from 1.5 to 20 per 1,000 live births.^5^ The incidence is comparatively higher when ultrasonography is employed in addition to clinical evaluation.^6^

In infants, DDH does not manifest any symptoms; however, as the youngster begins to walk, symptoms such as a limp, waddling gait, and discrepancy in leg length appear. These symptoms are typically painless. It is clinically irrelevant that the average age of walking lags behind by one month because there is a large range of walking ages and all of them are within the expected time. Pain and early osteoarthritis are among the late effects of undiscovered DDH, which frequently result in the need for premature hip replacement therapy. A restricted abduction of hip, a discrepancy in length of both legs, and an asymmetrical fold of buttock are examples of abnormalities that can be found during a physical examination.^5^

DDH is a fundamental reason of disability in children and young adults, making it a considerable socio-economic burden on the society. It is a preventable and treatable disorder. The use of ultrasound hip in newborns under one month of age carried out as early detection of DDH is the need of the day to avoid complications and surgical interventions. This study aimed to determine an association of ethnicity and gender with DDH in healthy newborns to promote early diagnosis.

## METHODS

This analytical cross-sectional observational study was carried out in Karachi between January and June 2022 after receiving ethical approval (ERC88/2021). Participants were included from Fazaia Ruth Pfau Medical College (FRPMC) and Bantava Anis Hospitals in Karachi.

**Fig.1:**
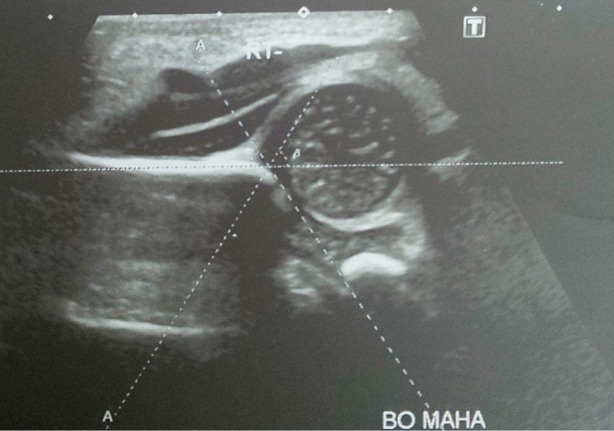
The alpha and beta angles measured on Graf’s Method Ultrasound.

Sample size of 115 was calculated using OpenEpi Software using 95% confidence interval. A total of 115 healthy newborns of both genders below the age of 28 days were enrolled in the study after parental consent. Infants with neuromuscular disease, neural tube defects or genetic abnormality were excluded from the study. The gender, ethnicity and hip angles were recorded in the proforma. A physical examination was then carried out to detect hip instability. Then, the hip joint angles were measured on ultrasound using Graf”s method (Fig.1).

**Fig.2:**
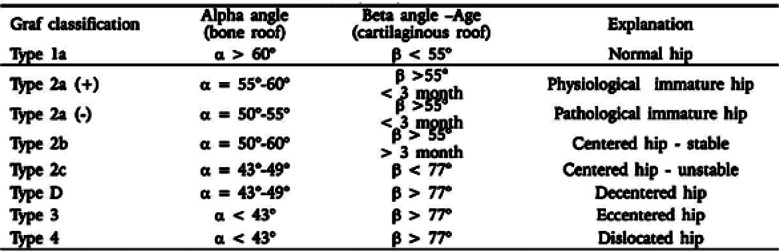
Graf’s Classification.

**Fig. 3:**
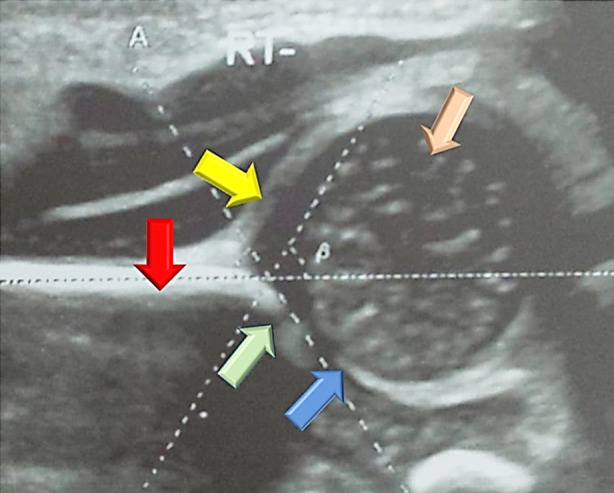
The Anatomical Landmarks used to measure Hip Angles on Graf’s Method Ultrasound indicated by arrows.

To determine the hip instability, the Ortolani and Barlow maneuvers were carried out by the Pediatric consultant.^7^ Ultrasound examination was done in coronal view using the Toshiba Aplio 300, High Frequency Linear Probe 7.5 MHz. The infant was placed in the lateral decubitus position and knees were flexed at 90º. Transducer was placed parallel and lateral to the hip. The alpha angle was measured between the bony acetabulum and the ilium. The beta angle was determined between the ilium and labrum.^7^ The hip angles were classified according to predetermined criteria by Cekic et al.^8^ (Fig.2). The anatomical landmarks used for the angle measurements were bony acetabular roof, labrum, iliac bone, triradiate cartilage and head of femur (Fig.3).

### Statistical Analysis:

The data was analyzed using SPSS version 23.0. The Chi-square and Pearson’s Chi-square tests were applied to determine frequency and significance of association. A p-value less than 0.05 was considered statistically significant.

## RESULTS

**Fig.4:**
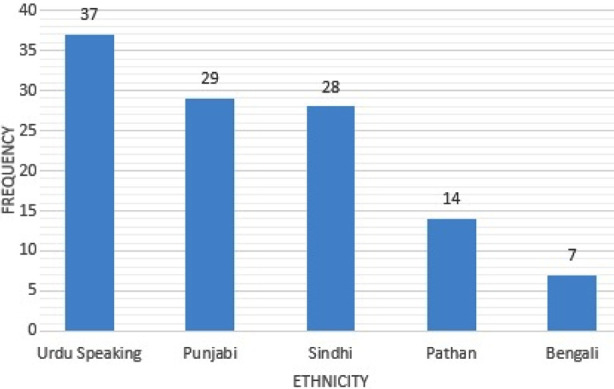
Distribution of Ethnicities in the Study Population (n=115).

The pathological hip was predominant in male newborns. The difference between gender distributions was not significant (Table-I). The study subjects belonged to five different “ethnicities” (Urdu Speaking, Punjabi, Sindhi, Pathan and Bengali) (Fig.4). The Urdu speaking group had the largest number of physiological immature hips as compared to pathological hips. An equal number of normal and pathological hips were found in the Pathan group. A high number of pathological hip was observed in the Sindhi group (Table-II). The difference between the groups was statistically significant (*p*-value= 0.021).

**Table-I:** Hip distribution according to the Graf method ultrasound according to Gender.

Graf type		Mature/physiological Immature hips (n, %)	Pathological Hips (n, %)	Total Hips	p-value
Gender	Male	81 (35.21%)	37 (16.08%)	118 (51.30%)	0.870
	Female	78 (33.91%)	34 (14.78%)	112 (48.69%)	
Total		159 (69.13%)	71 (30.86%)	230 (100.0%)	

p-value ≤ 0.05 is significant difference (*); Test applied: Chi- Square.

**Table-II:** Association between Ethnic groups and Pathological Immature/Pathological hips among the study population.

Ethnicity	Mature/physiological immature hips	Pathological immature/pathological hips	p -Value
Urdu speaking	59 (25.65%)	15 (6.52)	0.021*
Punjabi	41 (17.82%)	17 (7.39%)	
Sindhi	35 (15.21%)	21 (9.13%)	
Pathan	14 (6.08%)	14 (6.08%)	
Bengali	10 (4.35%)	4 (1.73%)	
Total	159 (69.13%)	71 (30.86%)	

p-value ≤ 0.05 is significant difference (*) *p-*value ≤ 0.001 is highly significant (**). Test applied: Pearson’s Chi- Square.

## DISCUSSION

It is estimated that 75% of DDH can be attributed to female sex alone, independent of any other known risk factors. This highlights the need to perform a thorough physical examination, especially Barlow and Ortolani maneuvers on every newborn to diagnose DDH.^9^,^10^ This could be because the maternal corpus luteum releases a hormone (relaxin) in early pregnancy that causes the ligaments of the hip joint to loosen, making the pelvis wider. Since females are more sensitive to relaxin hormone, this could cause female babies to develop “DDH”.^11^

In the current study, we observed more cases of DDH in male infants. However, we did not find a significant difference in gender distribution with the occurrence of DDH. One possible explanation for this could be the socioeconomic status of the study population with more value being given to male infants. A study in Saudi Arabia by Kardm et al.^12^ also observed male predominance for DDH. Shaikh et al.^13^ also reported high incidence of musculoskeletal anomalies including DDH in male infants in a hospital-based study in Larkana region of Sindh. Shaheen et al. also reported high incidence of musculoskeletal disorders in males.^14^ Similar to our study, Begum et al. 15 also reported higher number of pathological and immature hips in males as compared to females. Contradictory results were observed in several studies. Studies conducted in Turkey and Iran revealed a significantly higher proportion of female gender as compared to male gender in association with an increased risk of developmental dysplasia of the hip.^16^-^18^ Studies in Poland and England also reported female gender as a risk factor for DDH in infants.^19^,^20^

We observed significant association of DDH with ethnicity. Particularly among Sindhi, Punjabi, Urdu speaking and Pathan groups. The practice of tight swaddling of infants is common among Pathan and Sindhi ethnicities, which might be the cause. According to a hospital-based study, high incidence of musculoskeletal disorders was observed in the provinces of Punjab, Khyber Pakhtunkhwa, Azad Jammu Kashmir and Islamabad.^14^ Azmatullah et al. reported a high incidence of musculoskeletal disorders in Balochistan province of Pakistan. In the study, they observed DDH as the most common musculoskeletal disorder. Furthermore, they compared the Ethnicities according to the spoken language and observed high number of musculoskeletal disorders in the individuals from Pishin.^21^ A sizable Chinese cohort study undertaken by Li et al. revealed a high occurrence of the disease among their population.^22^ Similarly, a study carried out in Mexico revealed a strong association between ethnicity and hip dysplasia.^12^ Motta et al. 23 also observed high risk of DDH in relation to ethnicity (OR=2.561). High risk of DDH has been reported among Caucasian, Asian, Mediterranean and American populations. Genetic as well as environmental elements may also be responsible for this ethnic predilection in cases of DDH.^24^

## CONCLUSION

There is no significant difference in the incidence of DDH between male and female infants. DDH is prevalent in the Sindhi, Punjabi and Pathan populations. There is a strong association between these ethnicities and hip dysplasia in infants of 28 days age.

### Authors’ contribution:

**NM:** Contributed to the study design, questionnaire design, data interpretation.

**SKK:** Contributed to the literature search, and manuscript writing.

**AQ:** Contributed to the data analysis, data interpretation, critical review of article.

**MA:** Contributed to the questionnaire design, data collection and provided feedback through critical manuscript review.

All authors have read the final version and are responsible and accountable for the accuracy and integrity of the work.
